# 311. Factors Associated with Surgical Site Infection in Joint Replacement and Spine Surgery under a Staphylococcus Aureus Screening, Targeted Decolonization, and Restricted Vancomycin Protocol

**DOI:** 10.1093/ofid/ofaf695.107

**Published:** 2026-01-11

**Authors:** Jonathan K Pinsky, Mary Anderson, Zachary Pentony, Ryan Sullivan, Karen Murphy, Vishal Didwania, Jason Pinsky

**Affiliations:** Endeavor Edward Hospital Metro Infectious Disease Consultants, Naperville, IL; Edward Hospital, Naperville, IL, Naperville, Illinois; Endeavor Edward Hospital, Naperville, Illinois; Endeavor Medical Group, Naperville, Illinois; Endeavor Edward Hospital, Naperville, Illinois; Endeavor Elmhurst Hospital, Burr Ridge, Illinois; Northwell Health, Long Island Jewish Forrest Hills Hospital, Forrest Hills, New York

## Abstract

**Background:**

Screening and decolonization for *Staphylococcus aureus* (SA) reduces surgical site infections (SSI) in orthopedic surgery. A retrospective cohort study was performed to assess the risk associated with SA carriage under a targeted decolonization, and restricted vancomycin protocol in arthroplasty and spine surgery.

**Table 1:**
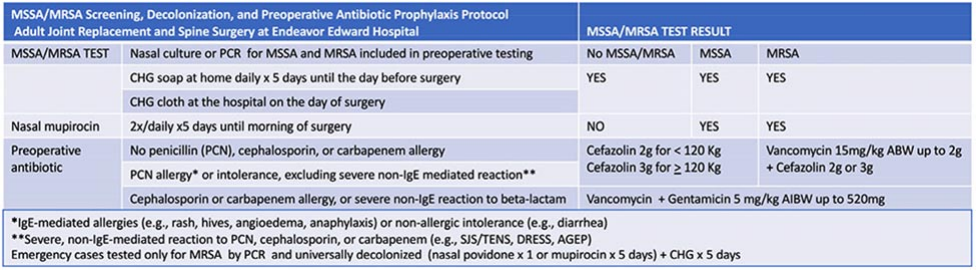
Endeavor Edward Hospital Adult MSSA/MRSA Screening, Decolonization, and Preoperative Antibiotic Protocol

**Table 2:**
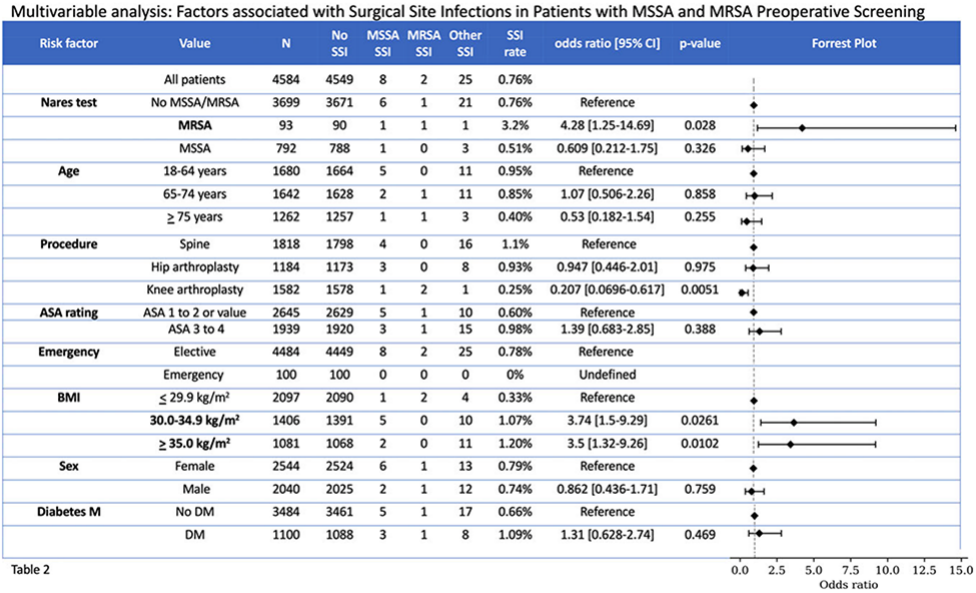
Risk factors and the associated: 1) number of procedures, 2) number of MSSA SSI, MRSA SSI, other SSI, 3) SSI rate, and 4) odds ratio for SSI

**Methods:**

Under facility policy (table 1) patients undergoing arthroplasty or spine surgery were tested preoperatively for methicillin-susceptible (MSSA) and methicillin-resistant (MRSA) SA by nasal culture or PCR. For five days before surgery, patients cleansed daily with chlorhexidine (CHG), and those who tested positive for MSSA or MRSA applied nasal mupirocin twice daily. Preoperative vancomycin was restricted to MRSA carriage or specific allergies. A retrospective cohort study was performed to assess the association of NHSN-defined SSI with MSSA carriage, MRSA carriage, body mass index (BMI), age, diabetes (DM), and American Society of Anesthesia rating (ASA). The cohort was adults who underwent knee or hip arthroplasty, spinal fusion, or laminectomies from 1/1/2022 to 6/30/2024, and who were tested for MSSA and MRSA < 39 days before surgery.

Tables 3 – 6

**Figure:**
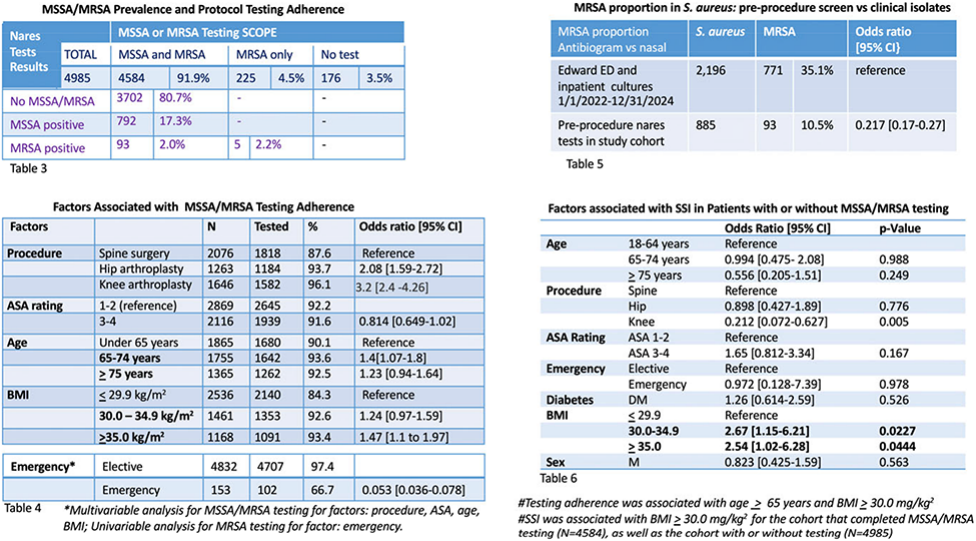

Table 3 (top left): Preoperative test adherence and MRSA/MSSA prevalence; Table 4 (bottom left): Factors associated with test adherence; Table 5 (top right): MRSA rate among Staph aureus isolates in preoperative nasal swabs compared to clinical isolates; Table 6(bottom right): MV analysis of factors associated with SSI with or without MRSA/MSSA testing

Tables 7 - 10

**Figure:**
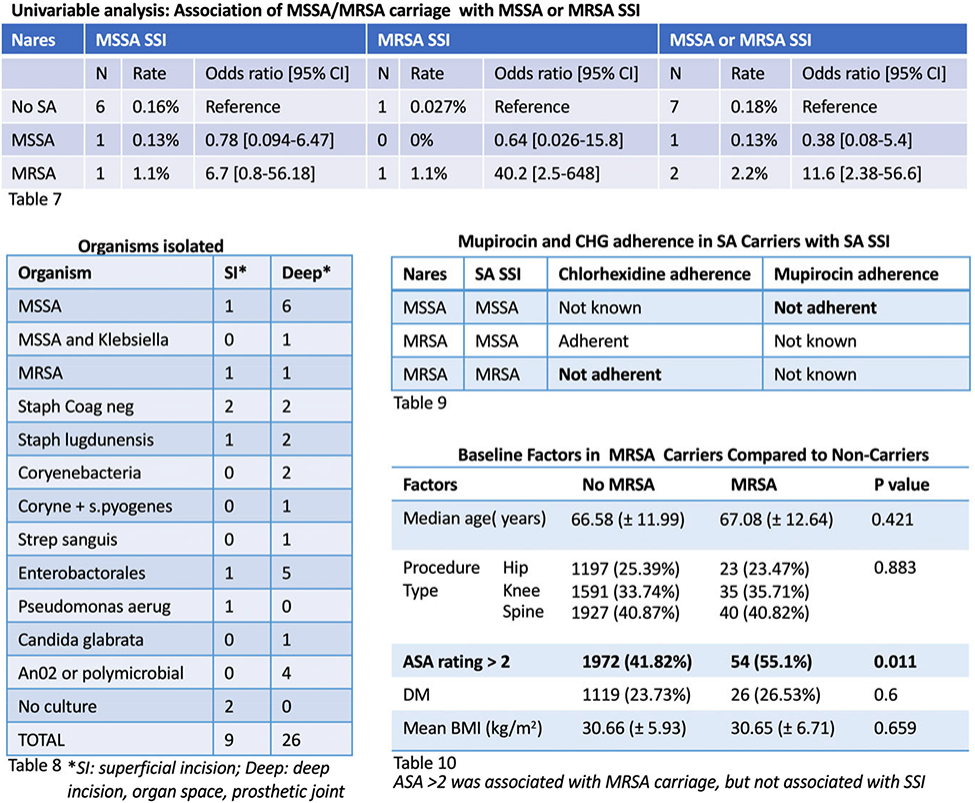

Table 7 (top panel): Univariate analysis of association with MSSA/MRSA carriage and MSSA/MRSA SSI; Table 8 (bottom left): Organisms isolated; Table 9 (middle right): CHG and mupirocin adherence in patients with MSSA/MRSA carriage who developed MSSA/MRSA SSIs; table 10 (bottom right): baseline factors in MRSA carriers compared to non carriers.

**Results:**

MSSA and MRSA testing occurred prior to 4,584 of 4,985 procedures. In 792 MSSA carriers, there was 1 MSSA SSI, no MRSA SSI, and 3 non SA SSIs. In 93 MRSA carriers, there was 1 MSSA SSI, 1 MRSA SSI, and 1 non SA SSI. In 3,699 non-carriers, there were 6 MSSA SSIs, 1 MRSA SSI, and 21 non SA SSIs. Nonadherence to CHG or mupirocin was documented in 2 of 3 SA carriers that developed SA SSIs (table 9). In a multivariable analysis (Table 2), factors associated with SSI (OR [95% CI]) were MRSA carriage (4.28 [1.25-14.7]), BMI 30.0-34.9 mg/kg^2^ (3.74 [1.5-9.29]), and BMI > 35.0 mg/kg^2^ (3.5 [1.32-9.26]). Factors not associated with SSI were MSSA carriage (0.61 [0.21-1.75), ASA > 2 (1.39 [ 0.68-2.85]), DM (1.31 [0.63-2.74]), male sex (0.86 [0.44-1.71]), age 65-74 years (1.07 [0.51-2.26]), and age > 75 years (0.53 [0.18-1.54]).

**Conclusion:**

Under a SA screening, targeted decolonization, and restricted vancomycin protocol in arthroplasty and spine surgery, SSI was associated with obesity and MRSA carriage, but not MSSA carriage. This suggests decolonization mitigated the risk related to MSSA carriage. MRSA SSI was rare in the absence of MRSA carriage.

**Disclosures:**

All Authors: No reported disclosures

